# Rotavirus Infection in Swine: Genotypic Diversity, Immune Responses, and Role of Gut Microbiome in Rotavirus Immunity

**DOI:** 10.3390/pathogens11101078

**Published:** 2022-09-22

**Authors:** Deepak Kumar, Frances K Shepherd, Nora L. Springer, Waithaka Mwangi, Douglas G. Marthaler

**Affiliations:** 1 Department of Diagnostic Medicine/Pathobiology, College of Veterinary Medicine, Kansas State University, Manhattan, KS 66506, USA; 2 Department of Microbiology and Immunology, University of Minnesota, Minneapolis, MN 55108, USA; 3 Clinical Pathology, Biomedical and Diagnostic Sciences, College of Veterinary Medicine, University of Tennessee, Knoxville, TN 37996, USA; 4 Indical Inc., 1317 Edgewater Dr #3722, Orlando, FL 32804, USA

**Keywords:** swine, rotavirus, immunity, genotypic diversity, lactogenic immunity, gut microbiome

## Abstract

Rotaviruses (RVs) are endemic in swine populations, and all swine herds certainly have a history of RV infection and circulation. Rotavirus A (RVA) and C (RVC) are the most common among all RV species reported in swine. RVA was considered most prevalent and pathogenic in swine; however, RVC has been emerging as a significant cause of enteritis in newborn piglets. RV eradication from swine herds is not practically achievable, hence producers’ mainly focus on minimizing the production impact of RV infections by reducing mortality and diarrhea. Since no intra-uterine passage of immunoglobulins occur in swine during gestation, newborn piglets are highly susceptible to RV infection at birth. Boosting lactogenic immunity in gilts by using vaccines and natural planned exposure (NPE) is currently the only way to prevent RV infections in piglets. RVs are highly diverse and multiple RV species have been reported from swine, which also contributes to the difficulties in preventing RV diarrhea in swine herds. Human RV-gut microbiome studies support a link between microbiome composition and oral RV immunogenicity. Such information is completely lacking for RVs in swine. It is not known how RV infection affects the functionality or structure of gut microbiome in swine. In this review, we provide a detailed overview of genotypic diversity of swine RVs, host-ranges, innate and adaptive immune responses to RVs, homotypic and heterotypic immunity to RVs, current methods used for RV management in swine herds, role of maternal immunity in piglet protection, and prospects of investigating swine gut microbiota in providing immunity against rotaviruses.

## 1. Rotavirus Genome, Classification and Host Range

Rotaviruses (RVs) are double-stranded RNA viruses belonging to the *Rotavirus* genus in the *Reoviridae* family. The RV genome is approximately 18,500 bp in size and consists of 11 segments of dsRNA encoding six structural proteins (VP1-VP4, VP6 and VP7) and five non-structural proteins (NSP 1- NSP5/6) [1]. The inner capsid of the virion comprises VP1, VP2, and VP3 segments. The VP6 forms the middle layer of the capsid, while the outer capsid is composed of the VP7 and VP4 proteins. VP7, a glycoprotein with a molecular weight of 37 kDa, constitutes 30% of the virus protein, and forms the smooth external surface of the outer shell. The minor component of the outer shell, VP4, is present as a series of spikes that project outward from the VP7 shell. VP4 is non-glycosylated, has a molecular weight of 88 kDa, and constitutes 1.5% of the virus protein [2]. Both VP7 and VP4 proteins independently induce neutralizing and protective antibodies [3]. The VP4 gets proteolytically cleaved into VP5* and VP8* segments. The VP8* forms the head of the VP4 protein which helps in host attachment and infectivity [4]. VP4 has been implicated in several important functions, including cell attachment and penetration, hemagglutination, neutralization, host range, and virulence [5]. RVs are unique since the NSP4 produces an enterotoxin, which contributes to viral pathogenesis [6].

RV species are classified based on sequencing of the VP6 gen 1 [7,8]. A binary classification system is used to address vast rotavirus diversity on the basis of sequencing of G (VP7) and P types (VP4). The dual (G/P) tying system has been extended to a complete genome classification system based on nucleotide sequencing of all 11 RV segments with nucleotide percent identity cut-off values set for each segment. In this system, VP7-VP4-VP6-VP1-VP2-VP3-NSP1-NSP2-NSP3-NSP4-NSP5/6 RV genes are designated as Gx-P[x]-Ix-Rx-Cx-Mx-Ax-Nx-Tx-Ex-Hx [9].

Currently, nine RV species (RVA-RVD and RVF-J) have been classified by the International Committee on Taxonomy of Viruses (https://ictv.global/report/chapter/sedoreoviridae/sedoreoviridae/rotavirus, accessed on 14 September 2022). However, only species A, B, C, E, and H have been reported from swine [10,11,12,13]. Humans and swine are affected by species A, B, C, and H RVs. Birds are affected by RV D, F, and G, and species E has been reported exclusively in swine. Host range of RVA includes humans [14,15], cows [16], goats [17,18], wild animals [19], ostriches [20], chicken [21,22], dogs [23], and horses [24,25]. RVB has been identified in pigs [10,26,27], cows [28,29], humans [30,31], goats, lambs [32], and rats. RVC has been detected from a variety of sources including pigs [27,33,34], humans [35,36,37], cows [38], cats [39], and dogs [40].

RVs are ubiquitous in nature, and mixed infections involving several RV strains appear more common as pigs grow older. Once infected, piglets may exhibit clinical or subclinical symptoms and eventual recovery in most cases. However, neonatal and suckling piglets without an established adaptive immune system are worst affected. RVA is the most characterized species among RVs due to its wide host range, high prevalence, and pathogenicity [10].

## 2. Rotavirus Entry and Replication

RV transmission is through fecal-oral route, and the piglets become infected with RV shed from sows and other piglets. The target sites of RV replication are the mature, non-dividing enterocytes in the small intestine. Enterocytes contain the enterokinase enzyme which is necessary for activating trypsin, which activates RVs and facilitates viral entry into the cells. The virus particularly affects the middle and the tip of the villi causing destruction and eventually resulting in villous atrophy [41,42]. The extent of villous atrophy induced by RV is lesser compared to other enteric viral pathogens of pigs such as transmissible gastroenteritis virus (TGEv) or porcine epidemic diarrhea virus (PEDv). The normal villus-height/crypt-depth ratio of the intestinal villi is approximately 7:1. TGEv reduces this ratio to 1:1, whereas RV infection slightly changes it to 5:1. The severity of RV infection depends on the length of the villi and the percentage of enterocytes that are affected. Eventually, the mature columnar epithelial cells on the villi are replaced by immature cuboidal enterocytes that are unable to produce digestive enzymes and have lost their absorptive capabilities [43].

**Figure 1:**
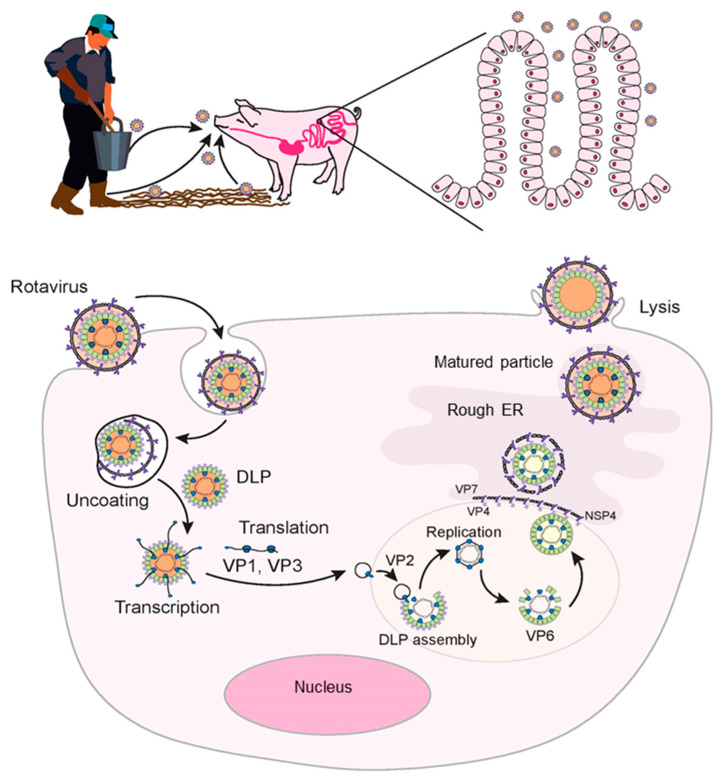
Rotavirus replication cycle. The rotavirus replicates in the cytoplasm of the enterocytes. Rotavirus enter the host cells by receptor-mediated endocytosis. The low calcium levels inside the endosome cause the removal of outer capsid layer, which results in the release of a double layered particle (DLP) into the cytoplasm. Viral mRNA is transcribed to form the structural proteins of the capsid. The RNA genome is replicated and packaged into newly made DLPs in viroplasms. Binding of DLP with NSP4 results in budding of DLPs into the endoplasmic reticulum (ER). In the ER, VP4 and VP7 proteins are added onto the DLPs thus forming a triple layered particle (TLP). The matured virions are released from cells through cell lysis.

The VP8* subunit of the VP4 binds to permissive enterocytes by interacting with sialic acid [44] or histo-blood group antigens (HBGAs) [45] on the cell surface, which is followed by interaction with other cell surface receptors such as integrins and heat shock cognate protein 70 (Hsc70) [45,46]. RV–HBGA interactions depend on the P genotype of the RV, not on the species of origin [47]. Sialoglycan ganglioside GM3 and GM1 serve as receptors for porcine RV strain OSU and human strains KUN and MO [48,49]. Specific VP4-HBGA interactions probably explain host range restriction among RVs. The virus is internalized into the cells by clathrin-dependent or clathrin-independent and caveolin-independent endocytic pathways [41,50]. The low calcium ion levels inside the endosome causes removal of the outer layer of virus particle and results in the formation of transcriptionally active double-layered particles (DLPs) into the cytoplasm [45] (Figure 1). Viral mRNA functions as a template for the production of viral proteins and genome replication. The replicated RNA assembles to form new DLPs in the form of viroplasms, which are specialized structures consisting of viral and cellular proteins. The freshly made DLPs then interact with NSP4, which facilitates entry of DLPs into the endoplasmic reticulum (ER). Moreover, NSP4 is also responsible for increased cytoplasmic calcium levels required for virus replication [51]. In the ER, outer capsid proteins VP7 and VP4 proteins are added to the enveloped virus particles which results in the loss of transient envelope and formation of triple layered particles (TLPs). The TLPs are then release from enterocytes through cell lysis (Figure 1).

## 3. Distribution and Genotypic Diversity of Porcine Rotaviruses

RVA has been reported from swine population globally. In the US, RVA infection is most common in pigs 21–55 days old, with slightly less neonatal cases [12]. However, RVA is still a major cause of neonatal diarrhea in piglets worldwide [52,53]. RVA prevalence ranging from 9.4% to 81.1% have been reported from swine populations in the US [12,26,34,54,55]. Till date, 42 G genotypes and 58 P genotypes have been identified within RVAs (https://rega.kuleuven.be/cev/viralmetagenomics/virus-classification/rcwg, accessed on 14 September 2022). Of these, 12 G genotypes (G1-G6, G8-G12, and G26) and 16 P genotypes (P[1], P[5]-P[8], P [11], P[13]-P[14], P[19], P[23], P[26]-P [27], P[32] and P[34]) have been reported from swine populations [56,57,58]. A study reported G5 (71.43%) as the most prevalent RVA genotype in the US followed by G4 (8.19%), G3 (3.57%), G9 (2.31%), G11 (1.89%), G10 (1.26%), and G1 (1.05%) [58]. Prevalence of other genotypes (G2, G6, and G10) was less than 1%. Among P genotypes, P[7] was the most common genotype (77.22%) followed by P[6] (12.07%), whereas other P types individually constituted less than 1% of the reported RVA genotypes [58]. Another study from the US reported G9P[13] as the most prevalent (60.9%) G and P type combination [54].

RVC has been emerging as a significant cause of enteritis in neonatal piglets [59]. Porcine RVC was first identified in 1980 and considered as an enteric pathogen with a moderate prevalence rate between 4 and 31% [60]. In the US, RVC is a major cause of diarrhea in neonatal pigs, particularly in piglets younger than 3 days old [61]. In the US, 51.1% of porcine intestinal samples collected during 2009–2011 were positive for RVC [12]. A study reported an overall RVC prevalence of 19.5% in diarrheic and non-diarrheic piglets collected from swine farms located in Ohio, US [62]. The prevalence of RVC was 23.5% among nursing piglets compared to only 8.5% in weaned piglets [62]. Another study from the US detected RVC in 46% samples of porcine origin (feces, fecal swabs, intestinal, or lung tissues) collected during 2009–2011 in the US and Canada [61]. Of these, RVC was detected in 16% of samples from very young pigs (<3 days old) and 21% of samples from young pigs (4–20 days old). Interestingly, 34% of RVC positive samples were negative for RVA/RVB, and the highest percentage of single RVC infections was in very young (78%, <3 days) and young pigs (65%, 4–22 days) compared to 6–39% in older age groups [61]. Interestingly, single infections of RVC are more common in 0–3 days old piglets, with co-infection with other RV species being more prevalent post-weaning [12,34].

In swine, 15 G genotypes (G12, G13, G8, G6, G5, G14, G9, G1, G17, G15, G7, G10, G3, G18, G16), and 16 P genotypes (P[1], P[5]-P[9], and P[12]-P[21]) of RVC have been identified [63,64]. The G6 genotype (70%) was the dominant RVC genotype followed by G5 (17%), G1 (12%), and G9 (1%). A study reported higher fecal prevalence (76.1%) of RVC from healthy and diarrheic piglets in the US [65]. A recent study reported presence of RVC in piglets less than 1-week-old in Australian swine herds [66]. Importantly, single RVC genotypes (either G5 or G6) were detected from neonatal piglets; however, older piglets (5–11 weeks) harbored multiple genotypes of RVC (G1 and G3) [66]. It is evident that RVC infections are more prevalent among neonatal piglets than weaned piglets, but the reason(s) are not completely understood. Likely reasons include lack of an RVC vaccine for use in swine, insufficient maternal RVC antibodies in colostrum or low minimum infectious dose of RVC required for infecting piglets compared to other swine enteric viruses [65].

Unlike RVA and RVC, Rotavirus B is more prevalent in older pigs and generally not considered an immediate cause of piglet mortality. Few studies have reported RVB prevalence from the United States [12,26]. A total of 31.8% diarrhea samples from pigs of North American origin were found positive for RVB [12]. A study reported 46.8% prevalence of RVB in pigs of all ages [26]. Most of the RVB positive intestinal samples (70/81) in this study also tested positive for RVA and RVC. The highest prevalence (72.7%) of RVB positive samples was observed in pigs more than 55 days of age compared to only 12.9% RVB positive samples below 21 days of age [26]. A study from Japan reported 25.9% prevalence of RVB in pigs [67]. Age distribution revealed 71.9% RVB positivity in diarrheal fecal samples from weaned pigs compared to 18.7% in diarrheal feces from suckling piglets. RVA and RVC were detected in 36.4% and 21.2% fecal samples, respectively [67]. Despite having high detection rates in swine, pathogenesis of RVB has been scarcely researched [10,32]. A recent study successfully reproduced clinical illness in 10-days-old piglets experimentally inoculated with fecal suspension collected from RVB positive diarrheic piglets [10]. The fecal samples were negative for common swine viral pathogens including RVA, and the presence of RVB was confirmed by next generation sequencing (NGS). The inoculated piglet developed diarrhea within 12 h of inoculation, and NGS of intestinal homogenate identified RVB. Of the 26 G genotypes and 5 P RVB genotypes known in all host species, 21 G genotypes (G4 and G6-G26) and 2 P genotypes (P[4]-P[5]) have been identified in pigs [68].

There is only a single report of RVE in swine identified in the 1980s, and the sample is no longer available [11]. There have been no reports of RVE since then to accurately analyze its host specificities and epidemiology. RVH was recently proposed and included three human strains (ADRV-N, J19 from China and B219 from Bangladesh) and a porcine RVH strain SKA-1 isolated from a pig with diarrhea in Japan [69,70,71,72,73]. In 2012, three more porcine RVH strains BR63, BR60, and BR59 were reported from Brazil [74]. In the same year, 15% of porcine fecal samples comprising different age groups were positive for RVH in the US [13]. Of the RVH positive samples, 18% were detected in 21–55-days-old pigs; however, no RVH was detected in 1–3-day-old piglets. Phylogenetic analysis revealed that the RVH had been circulating in US swine herds at least since 2002 and had remained underdiagnosed [13]. Recently, 12 porcine RVH strains from Japan were sequenced and genotype constellations were allotted [64]. A total of 10G, 6P, 6I, 3R, 4C, 7M, 6A, 2N, 4T, 6E and 3H representing VP7, VP4, VP6, VP1, VP2, VP3, NSP1, NSP2, NSP3 genes were identified. Most common G and P genotypes were G5 and P1, respectively [64].

Overall, huge genetic diversity exists among porcine RVs circulating within the swine herds. Hence, sequencing based routine surveillance of RV genotypes in the swine herds is of utmost importance to identify novel emerging RV strains. Emerging RV G and P genotypes could be quickly included in the vaccines to limit their further dissemination.

## 4. Immune Responses to Rotavirus Infection

The innate and adaptive immune responses play a key role in containing RV infection in infected hosts [75]. T-lymphocytes, mediating cellular immunity, along with B lymphocytes, mediating humoral immunity, provide the adaptive immune response, which works in close association with the innate immune system.

### 4.1. Innate Immune Response

#### 4.1.1. Role of RIG-I-like Receptors

Innate immune response is the combination of the host’s non-specific defense mechanisms critical for early pathogen recognition and inhibition [76]. Different effectors of the innate immune response include macrophages, dendritic cells (DCs), natural killer cells (NKs), chemokines, and various cytokines such as interleukins, and interferons (IL and IFN) [77,78]. The initiation of the immune response to an invading microorganism like a virus require that the host senses the organism and its constituents. The initial response is carried out primarily by pattern recognition receptors (PRRs), which are expressed by intestinal epithelial cells and recognize the conserved molecular footprint of pathogens called pathogen- or damage-associated molecular patterns (PAMPs and DAMPs) [79,80].

The presence of viral sensing PRRs in multiple cellular compartments allows innate cells to recognize and quickly respond to a broad range of viruses. RV dsRNA triggers cytoplasmic PRRs such as RIG-I-like receptors RIG-I (Retinoic acid-inducible gene I), MDA5 (Melanoma Differentiation-Associated protein 5), LGP2 (laboratory of genetics and physiology 2), and endosomal membrane-associated PRRs such as toll-like receptor (TLR) 3 expressed within intestinal epithelial cells (IECs) and DCs [81,82]. TLR2, TLR5, and TLR7 have also been implicated in the innate immune signaling of RVs [83,84]. Both RIG-I and MDA5 recognize different sections of the same viral genome due to their preferential binding to RNA, which illustrates their ability to work independently and synergistically [76,85]. This is particularly true in viral infections such as RV, in which both of these receptors are required to induce the necessary levels of IFN-β signaling to control infection [76,81]. 

Upon binding to dsRNA, the activated RIG-I and MDA5 interact with the mitochondrial antiviral signaling proteins (MAVS) and form protein complex containing several different proteins [76,86]. Infection of porcine IECs by RVs trigger TLR3, RIG-I, and MDA-5 which then activates IRF3 and nuclear factor-κB (NF-κB), and induces expression of IFN-stimulated genes (ISG) [87]. A study using siRNA silencing in human IECs suggested that RIG-I and MDA-5 are more important for virus recognition and signaling for IFN production compared to TLR3 and dsRNA-activated protein kinase (PKR) [81]. Silencing RIG-I or MDA5, or MAVS, significantly decreased IFN-β production and increased RV titers in infected IECs. RV-infected mice lacking TLR3 or PKR did not change the levels of IFN-β and amount of RV in intestinal epithelium and feces. A study in suckling mice showed that both type I and type III IFNs are required to protect the gastrointestinal tract against the heterologous simian RV infection [88]. Moreover, both IFN types were demonstrated to independently contribute to innate antiviral defenses within the intestinal mucosa and cooperate to restrict extra-intestinal RV replication in other tissues [88].

Another member of the of RIG-I like receptors family, LGP2, appears to support RV replication, unlike RIG-I and MDA5 [81]. LGP2 receptors lack CARD domains (caspase-recruitment domains) found in RIG-I and MDA5 and hence cannot utilize MAVS signaling pathway [89,90]. LGP2 has a dual role of a negative and positive regulator of RIG-I/MDA5 signaling. It negatively regulates RIG-I/MDA5 signaling by competing with these receptors for binding with RV RNA. Overexpression of LGP2 has been linked with decreased IFN-β production, decreases IFN-sensitive response element (ISRE) activation, and increased RV titers in RV infected IECs [81].

#### 4.1.2. Role of Toll-like Receptors (TLRs)

Among Toll-like receptors, TLR3 is the most extensively researched receptor in RV infection. All TLRs, except TLR10, have been detected in primary IECs, however, their role in protection against RV infection is controversial [91]. RV dsRNA and its synthetic analog polyinosinic-polycytidylic acid poly(I:C) induce severe mucosal damage via TLR3-dependent manner [92]. Upon interacting with TLR3 within IECs, RV dsRNA stimulates the secretion of IL-15 which further increases the production of CD3+/NK1.1+ intestinal intraepithelial lymphocytes (IELs), a cell-type vital in maintaining the integrity of mucosal immune responses [93]. The enhanced cytotoxicity of IELs results in disrupted epithelial homeostasis and acute RV gastroenteritis indicating that TLR3 pathways have a role in RV pathogenesis [92].

Another study showed that RV dsRNA induces severe apoptosis and regression of wound repair in rat IEC-6 cells through a TLR3 dependent manner [94]. Anti-TLR3 antibodies reduced apoptosis and increased wound repair. RV recognition by TLR3 and increased TLR3-mediated pathogenesis is linked with age-dependent expression of TLR3 [95]. TLR3 expression was reported to be very low in the epithelium of suckling mice but strongly increased during the postnatal period. Increased postnatal TLR3 expression positively correlated with decreased RV susceptibility, viral shedding, and histological damage. The age dependent TLR3 upregulation was also found in human small intestinal biopsies [95]. Differences in TLR3 expression perhaps explains the high severity of RV infection in infants and young children (low TL3 expression) and better protection in adults (high TLR3 expression).

The role of other TLRs and proteins in the TLR pathway in RV clearance has also been explored. A study found that the absence of MyD88 signaling protein results in higher RV infectivity in a mouse model indicated by high RV shedding in feces, intestinal lysates, and high levels of virus in blood [96]. Loss of MyD88 also affected the humoral immune response, as evidenced by low RV-specific IgA and RV-specific IgG2c/IgG1 ratios. Since MyD88 mediates signaling for all TLRs, except TLR3, it is apparent that TLRs other than TLR3 also play a pivotal role in development of both innate and adaptive immune responses to RVs. Bacterial flagellin has been reported to prevent and cure RV infection in mice via TLR5 and NOD-like receptor C4 (NLRC4), receptors for bacterial flagellin [84]. Flagellin-induced activation of TLR5 and NLRC4 resulted in the production of the IL-22 and IL-18, respectively. Interestingly, administration of IL-22 and IL-18 to mice fully recapitulated the capacity of flagellin to prevent or eliminate RV infection. Flagellin’s protection against RV infection was eliminated in the absence of both TLR5 and NLRC4 or MyD88, which is required for signaling by TLR5 and inflammasome-associated cytokines [84].

Recently, a novel inflammasome sensor NLRP9b was recognized to have a role in RV dsRNA sensing [97]. Targeted deletion of NLRP9b, a NOD-like receptor in IECs of suckling mice resulted in increased diarrhea and RV shedding in feces compared to wild-type mice, illustrating a vital role of NLRP9b in RV infection. Intestinal organoids lacking NLRP9b also illustrated defective pyroptosis and decreased IL-18 production [97].

#### 4.1.3. Other Mediators of Innate Immune Response

Other innate immune cells involved in controlling RV infection include macrophages and DCs. DCs are considered the link between innate and the adaptive immune responses [98]. DCs are the most efficient antigen-presenting cells and play a vital role in the initiation of innate immune response against viral infections [99]. RV present in the intestinal lumen are transported to the Peyer’s patches (PPs) by M cells [100]. Viral antigen is then captured by DCs which results in upregulation of CD40, CD80, and CD86 surface activation markers [99]. During RV infection, DCs also effectively present viral antigens to T-cells. In RV-infected mice, a two-fold increase in the absolute numbers of DCs and the upregulation of surface activation markers CD40, CD80, and CD86 were observed compared to mice infected with UV-inactivated RV [99].

In vitro studies have shown that macrophages use MAVS to produce IFN-β and IL-6 in response to RV infection [101]. Knocking out upstream signaling by MDA-5 and RIG-I showed that only RIG-I seems to be important for anti-RV signaling in macrophages. In intestinal lymphoid tissues, DCs seemed to be responsible for the higher levels of observed IFN-α production. Increasing the dosage of RV inoculum in gnotobiotic pigs did not change the levels of IFN-α produced, suggesting that host cells are able to inhibit IFN production above a certain concentration to limit the amount of intestinal damage caused by inflammation [102].

Tumor necrosis factor alpha (TNF-α) is a multifunctional cytokine that has a potent antiviral role against influenza [103,104], hepatitis C [105], African swine fever virus [106], and RV [75]. Anti-RV effects of TNF-α are independent of IFN production and JAK-STAT signaling pathways [75]. Instead, TNF-α was reported to signal through the classical NF-κB pathway to inhibit RV infection [75]. Use of TNF-α inhibitors such as infliximab, which bind specifically to TNF-α and blocks its interaction with TNF receptors, completely blocked the inhibitory effects of TNF-α. A significant increase in levels of TNF-α has been reported in RV-infected children with fever and more episodes of diarrhea than those without fever and with fewer episodes of diarrhea. Although the mechanism behind the increase in levels of TNF-α is not completely understood, the authors posited that TNF-α induces increased levels of chloride ion secretion in IECs causing fluid loss from cells [107].

### 4.2. Adaptive Immune Response

Although the innate immune response against RVs is important, adaptive immune responses ensure efficient viral clearance and protection from re-infection. Several studies using mice and gnotobiotic piglets deficient in different arms of the immune system have been crucial to understanding the role of innate and adaptive immunity in clearance of and resistance to RV infections. Mice without T or B cells develop chronic infections of RV, and the lack of B cells greatly affects their ability to develop resistance in upon repeated exposure [108]. Mice without B cells eventually clear RV infection, although infection occurs earlier after exposure and lasts several days longer [108,109]. Not surprisingly, this illustrates a multi-level and coordinated approach of all arms of the immune system to clear RV infection. In the absence of humoral immunity, cytotoxic T lymphocytes can clear infection, but these populations of T cells are usually short-lived and cannot confer long-term immunity [109].

During RV infection, antibodies are produced against VP7, VP4, VP6, NSP3, and NSP4 [110,111,112,113,114,115]. However, the nature of immune responses to these proteins vary, and only VP7 and VP4 stimulate neutralizing antibody responses. Although the number of intestinal IgA-specific antibody secreting cells (ASCs) have been considered a strong indicator of protective immunity, routine quantification of ASCs is not feasible and hence serum IgA titers are used as an indicator of protection [110,116,117]. Studies in mice [108,118] and gnotobiotic piglets [119,120] have looked into the relative roles of B and T cells in active immunity against RVs. To understand the roles of B and T cells in RV immunity, B cell-deficient pigs, CD8 T cell-depleted pigs, and wild-type (WT) pigs were vaccinated with an attenuated HRV (human rotavirus) vaccine and challenged with virulent HRV [120]. B cell-deficient pigs experienced significantly longer duration of virus shedding compared to WT pigs, emphasizing the importance of B cells in vaccine-induced protective immunity [120]. Moreover, vaccinated B cell deficient and CD8 T cell-deficient pigs shed significantly higher titers of RV than WT pigs and CD8 T cell sufficient pigs, stressing the importance of CD8 T cells in containing RV replication. Therefore, both B cells and CD8 T cells play an important role in the protection against RV infection [118,120].

There is no cross protection between different RV species. Cross-protection against multiple genotypes of the same RV species (heterotypic protection) is an important component of the protective immune response against RVs in humans [121]. Human studies suggest that immunization with a single strain of RV provides substantial protection from severe infection caused by other RV strains [122,123]. Monovalent human RV vaccine containing G1P[8] induces significant protection against severe RV disease caused by multiple G and P types not included in the vaccine, which confirms at least some level of heterotypic protection from other G and P types. Although the mechanisms and the antigenic determinants underlying the heterotypic protection are not well understood, the presence of antibodies against non-neutralizing cross-reactive VP7 and VP4 epitopes, or VP6 have been suggested [124]. Natural infection or vaccination results in mainly homotypic RV immunity mediated by antibodies against VP7 and VP4, whereas previously exposed or adult animals produce homotypic as well as antibodies to a wide range of heterotypic RVs [125].

Studies in swine provide evidence of heterotypic protection against RV genotypes [126,127]. Inoculation of piglets with a porcine RVA (PRV) G9P[13] genotype provided complete (100%) short-term protection against homologous (PRV G9P[13]) and heterologous (human RV Wa G1P[8], HRV) challenges as evidenced by no viral fecal shedding and diarrhea in both challenge groups [127]. However, piglets inoculated with HRV Wa G1P[8] could prevent shedding and diarrhea in 16.6% and 66.7% piglets respectively, when challenged with heterologous PRV G9P[13]) strain. It was also revealed that PRV G9P[13] induced low levels of cross-neutralizing antibodies against selected porcine (OSU G5P[7] and Gottfried G4P[6]) and human (Wa G1P[8]) RVs [127]. Based on this finding, authors concluded that that heterologous protection against human Wa G1P[8] was not dependent on the heterotypic serum virus neutralization titers, and factors such as upregulated innate, mucosal, or cellular immune response might be responsible for heterotypic protection. It is important to note that piglets were not challenged with porcine OSU G5P[7] and Gottfried G4P[6] strains to asses heterotypic protection against these porcine genotypes [127]. 

Another study reported that antiserum to porcine RVA A2 strain with a G9P[9] genotype (previously identified as a G4P[7] strain) significantly neutralizes different human G9 strains in vitro, including 116E, R44, R143, US1205, INL1, and BD524 originating from different countries [126]. Antiserum generated against each of these human G9 strains also neutralized porcine A2 strain significantly. It was reported that the VP7 of the porcine A2 strain is similar to that of phylogenetic lineage 3 of human RVA G9 strains and also share amino acid substitutions with lineage 3 human G9 strains [126]. 

A study reported that infection-induced heterotypic immunoglobulins (Igs) mainly target VP5* region of VP4 [113]. Heterotypic protective Igs against VP7, and VP8*, are also generated after infection; however, homotypic anti-VP7 and non-neutralizing VP8* responses are more common [113]. These results specifically outline the importance of the VP5* region in mediating broad-based protection against serotypically distinct RV strains. Interestingly, the authors found that all VP8* specific monoclonal antibodies were inactive in traditional neutralization assay using MA104 cells and did not prevent RV associated diarrhea in mice, which was unusual. In a recent publication from the same group, the authors reexamined the ability of monoclonal antibodies (*n* = 32) to neutralize RVs in human IECs, including ileal enteroids and HT-29 cells [128]. Most (18 of 20) of the “non-neutralizing” VP8* mAbs efficiently neutralized human RV in HT-29 cells or enteroids. VP8* monoclonal antibodies also protected suckling mice from diarrhea in an in vivo challenge model. Authors concluded that since MA104 cells are the most commonly used cell line to detect anti-RV neutralization activity, previous studies might have underestimated the contribution of VP8* antibodies to the neutralization titer [128].

VP6 has also been explored as a vaccine candidate. Anti-VP6 IgA antibody delivered in a “backpack tumor model” were able to prevent primary and resolve chronic RV infections in a mouse model [129]. This same effect was not seen when the anti-VP6 IgA antibodies were injected directly into the lumen, suggesting that the main mechanism of protection involves the transcytosis of anti-VP6 IgA. A full, triple-layered RV particle is not transcriptionally active due to a conformational change during VP7 and VP6 interaction which decreases the activity of the VP1 polymerase. Researchers used this knowledge to discover that anti-VP6 monoclonal antibodies would interact with VP6 in a similar manner to the VP7 protein to stop viral transcription and replication. The immune system response in a mouse model after immunization with VP6 required the presence of αβ CD4 T cells, rather than γδ T cells or B cells [130]. 

There is a scarcity of data on immune responses to porcine RVs. Available data on adaptive immune response (humoral and cell mediated) to RVs in swine stems from the studies where gnotobiotic piglets were used as a model to study immune responses to human rotaviruses. There is a necessity to generate adaptive immunity data against porcine RVs to understand the dynamics of antibody-based and T-cell-based immunity against RVs, correlates of protection against RVs, the effects of co-infection of different RV species in mediating immunity against each other, and the level of cross protection conferred by different RV G and P-types.

## 5. Maternal Immunity and Protection of Piglets

Since piglets get infected with RV at birth, it is impractical to vaccinate the piglets to the RV field strains. The best approach is to immunize gilts before farrowing to boost their antibody levels, which can be passively transferred to the piglets through lacteal secretions. Boosting the lactogenic immunity appears to be the most efficient way of providing RV immunity to the piglets until the piglets reach an age at which they are less susceptible to rotaviral infections. Passive immune protection occurs in the form of high IgG antibodies in colostrum and high secretory IgA (sIgA) antibodies in colostrum and milk. In particular, sIgA antibodies play a major role in preventing RV infection at the gut mucosal level [131]. IgG and IgA produced in the sow traffics to the mammary glands and is transferred through colostrum and milk to piglets, where RVs are locally neutralized in the gut [65,132,133]. Since lactogenic IgA has high affinity and is more resistant to proteolytic degradation, it is more effective within the intestinal tract of the neonatal piglets [134]

IgA is the longest-lasting Ig present in lactating sows, but antibodies are typically strongly protective for only two weeks after farrowing [135]. Levels of IgG and IgM in piglets also fade over time, until active immunity is induced in the piglets resulting in an increase in anti-RV neutralizing antibodies [136]. Early weaning and lack of colostrum results in severe RV diarrhea in piglets, signifying the importance of colostrum antibodies in providing protection to piglets [137,138]. IgA levels in milk plays a vital role in lactogenic immunity and RV passive protection in suckling piglets. Studies from other swine enteric viruses have also identified IgA as an important correlate of passive immunity to piglets. An increased rate of protection against TGEv in neonatal piglets was associated with high sIgA levels in colostrum and milk [138].

Two modified live virus (MLV) vaccines, ProSytstems Rota and ProSystems RCE, developed by Merck Animal Health, are commercially available for use against RVA in swine. However, field testing and usage data for both of these vaccines is scarce and hence a true estimate of their efficacy cannot be established. Diversity of RVA strains other than the vaccine strains co-circulating in swine farms may assist RVAs to escape vaccine-derived immunity. Despite being the most common cause of rotaviral diarrhea in piglets less than 1 weeks of age, no vaccine is available for RVC due to challenges of adapting RVC to cell culture. However, recently a vectored vaccine platform known as Sequivity has been introduced by Merck animal health for use in pre-farrow gilts/sows against RVs. Sequivity is an RNA particle (RP) vaccine based on farm-specific VP7 sequences of RVA and RVC. Early trials of the Sequivity RP vaccine for RVs showed that RP vaccine yielded lower mortality and higher weight gain than natural planned exposure (NPE) [139], but more research is required to understand its effectiveness in providing protection against RVC in swine.

Lack of updated strains in the current RVA vaccine and absence of modified live virus (MLV) vaccines against RVC have prompted swine producers to mimic natural RV infection in gilts or sows in the form of “feedback” or “natural planned exposure (NPE)”, which contains RV-infected material. Gilts or sows are fed NPE to stimulate maternal immunity and to provide lactogenic immunity to piglets. NPE method involves feeding live farm-specific RV strains mixed with feed a few weeks prior to farrowing. Using NPE precludes the need to generate RVB and RVC in vitro and hence is currently the only method available to prevent RVB and RVC infections in swine farms. However, there are some disadvantages of using NPE at swine farms. One of the major concerns is the possibility of introducing unwanted viral and bacterial pathogens in the farrowing room. Stimulating high levels of passive immunity without introducing RV particles into the farrowing room is difficult since sows can have subclinical RV infections yet shed high amounts of virus into the environment. An optimal NPE dosing strategy has not been determined or standardized across the industry, making it difficult to know what strategies may be most effective in production settings. Another major challenge to create NPE material with high RVC viral load. Attempts have been made to determine the best time for exposing sows to the feedback material. NPE at 5, 4, and 3 weeks prior to farrowing is considered most successful in preventing pre-weaning RVA shedding in piglets. Three doses of NPE induced the highest level of RVA and RVC shedding in gilts following NPE and led to the least shedding and best performance in piglets [140]. However, some sows still shed RVA and RVC in the farrowing room, potentially exposing their piglets to higher levels of RVs.

There are several areas of future research in the field of immunity against porcine rotaviruses, which include: (1). Use of swine enteroids to propagate RVC and other difficult to grow RVs. Successful propagation of RVC will pave the way for the development of modified live virus (MLV) vaccines against RVC; (2). Although NPE is the most widely practiced method to stimulate maternal against RVA and RVC, lack of serological assays to assess antibody response to NPE or natural RV infection makes it difficult to assess true efficacy of NPE protocols. Hence, it is recommended to develop genotype-specific RVA and RVC-specific indirect ELISAs to measure antibody response to RVs in swine; (3). Finally, NPE material administered to gilts prior to farrowing contains both RVA and RVC genotypes. However, we do not know whether both of these viruses affect each other’s ability to colonize swine gut, and hence results in varied immune response. It would be interesting to investigate the differences in gut colonization capability and antibody responses to RVA and RVC in an individual and co-infection model.

## 6. Gut Microbiome and Rotavirus Immunity

### 6.1. Composition of Swine Gut Microbiome

The gut microbiota helps maintain normal functioning of the intestinal mucosal barrier and stimulate host immune response. Recent studies suggest that gut microbiota also play a crucial role in the regulation, elimination, and potentiation of infectious diseases. In pigs, Bacteroidetes and Firmicutes are predominant phyla of gut microbiota regardless of age and breed [141]. Normal gut microbiota of 4- to 21-day-old piglets includes Firmicutes (44%), Bacteroidetes (21%), Verrucomicrobia (20%), Proteobacteria (10%), and Fusobacteria (5%) [142]. Microbiome-virus interactions have been well characterized for Porcine Epidemic Diarrhea virus (PEDv), another important enteric viral pathogen of pigs [142,143,144,145,146]. However, such information is completely lacking for RVs in pigs. Moreover, association between gut microbiome changes in pigs and immune response to RVs has not been explored yet. A recent study tracking the pig microbiome from day zero until the market age also found Firmicutes to be the most abundant phylum followed by Bacteroidetes across each stage. These two phyla accounted for 70% of the total sequences [147].

### 6.2. Evidence from Human Rotavirus Studies

Recently, changes in gut microbiome composition have been correlated with improved protection against viral diseases such as porcine epidemic diarrhea virus (PEDv) [144], human RV [148,149], and porcine circovirus [150]. Much of our current understanding of association between RV immune response and gut microbiome stems from studies carried out in human infants within the last 5–6 years [148,151,152,153]. Human RV studies show that changes in gut microbiome composition are associated with improved immune response to RV vaccines [148,152]. In humans, RV vaccine immunogenicity correlated with an increased abundance of specific Proteobacteria (*Escherichia coli* and Serratia) in Pakistan and an increased abundance of *Streptococcus bovis* and decreased abundance of Bacteroidetes in Ghana [148,152]. In both the studies, pre-vaccination intestinal microbiome of infants differed significantly between RV vaccine responders (post-vaccination serum IgA titer > 20 IU/mL) and non-responders (post-vaccination IgA titer < 20 IU/mL). Interestingly, microbiome composition of vaccine responders was more similar to age-matched healthy Dutch infants, which further strengthens the important role of the gut microbiome in shaping immune response to RVs. Proteobacteria in particular stimulates immune responses through their expression of flagella or toxigenic LPS. A study from India reported no significant differences in microbiome diversity, stability, and taxon abundance between RV vaccine responders and non-responders [153]. The poor seroconversion (31%) in this study was presumed to be due to the presence of a specific bacterial community inhibitory to RV replication. 

### 6.3. Role of Probiotics and Vitamin A in Immunity against Rotaviruses

Probiotics have been used to improve immune response to RV vaccines in humans with varied success [153]. *Bifidobacterium* and *Lactobacillus* spp. were found to significantly reduce the duration of RV induced diarrhea in infants [154]. Both probiotics also appeared to ease the duration of fever, frequency of diarrhea, and vomiting; although, the association was not statistically significant [154]. Rice bran, a prebiotic, provided complete protection against human RV-induced diarrhea in *Lactobacillus rhamnosus* GG (LGG) and *Escherichia coli* Nissle (EcN) colonized gnotobiotic pigs [155]. Rice bran significantly enhanced the growth and colonization of both LGG and EcN in the intestine of pigs, promoted body weight gain, protected against damage to intestinal epithelium, and significantly enhanced intestinal IFN-γ and IgA levels compared to the non-rice bran group [155]. *E. coli* Nissle 1917 (EcN) was reported to mediate enhanced protection against human rotavirus (HRV) compared to *Lactobacillus rhamnosus* GG (LGG) in a gnotobiotic piglet model [156]. In this study, EcN colonized gnotobiotic piglets when challenged with HRV, resulted in increased plasmacytoid dendritic cells (pDCs), significantly enhanced NK-cell function, and reduced frequencies of apoptotic and TLR4+ mononuclear cells when compared to noncolonized, EcN negative, LGG negative, and EcN- and LGG-administered piglets [156]. A combination treatment including LGG and anti-RV antibodies significantly reduced RV induced diarrhea, prevented histopathological changes, and reduced the viral load in the intestines in mice [157]. In contrast to the studies demonstrating positive effects of probiotics, zinc and probiotic supplementation did not significantly improve the low immunogenicity of RV vaccine given to infants in a poor urban community in India [158]. In another study, dietary intake of *Bifidobacterium lactis* and *Streptococcus thermophilus* failed to decrease the duration of RV-induced diarrhea in infants [159]. The therapeutic ability of probiotic compounds is mainly attributed to their ability to reinforce the intestinal mucosal barrier, production of antimicrobial compounds, and stimulation of gut-specific immune response [157]. 

Few studies have investigated an association between Vitamin A and immune response to human rotavirus vaccines in gnotobiotic piglet model [160,161,162]. It has been reported that vitamin A deficient gnotobiotic piglets (VAD, born to VAD sows) immunized with human RV vaccine (RotaTeq) and later challenged with virulent G1P[8] human rotavirus (HRV), shed 350-fold more RV in feces compared to the piglets vitamin A sufficient piglets (VAS, born to VAS sows) [161]. Post HRV challenge, the intestinal HRV IgA titers were 11-fold lower in immunized VAD piglets compared to immunized VAS piglets. In a similar study, VAD and VAS piglets with or without vitamin A supplementation were orally immunized with attenuated HRV and later challenged with virulent HRV [160]. It was reported that immunized VAD piglets had lower serum IgA HRV levels and significantly lower intestinal IgA antibody secreting cells (ASCs) post-challenge, which also resulted in higher diarrhea severity scores in the immunized VAD piglets [160]. It is evident vitamin A deficiency impairs the ability of piglets to mount an efficient immune response experimental human rotavirus infection.

### 6.4. Gut Microbiome Modulation and Response to Rotavirus Infection

The probiotic based studies have failed to address the key question of whether there is a causal association between gut microbiome and RV vaccine immune response. Recently, malnutrition was suggested to reduce the protective efficacy of oral live attenuated human RV vaccine (attHRV) in human infant fecal microbiota (HIFM) gnotobiotic piglet challenge model [163]. Four groups of gnotobiotic piglets were fed either sufficient (with and without HIFM) or deficient diets (with and without HIFM). Pigs in deficient HIFM and sufficient HIFM groups were orally inoculated with 2 mL of diluted HIFM stock at 4 days of age. All pigs were given oral attenuated HRV vaccine twice after fecal transplantation at post-transplant day (PTD) 7 and 17, subsequently challenged with virulent HRV and euthanized at PTD31. Piglets fed deficient diets had reduced HRV-specific IgG and IgA ASCs in blood or intestinal tissues following AttHRV vaccination and before virulent HRV challenge [163]. Few studies have reported the effect of nutritional supplements in enhancing immune response to swine viral pathogens [164,165].

Gut microbiome modulation using narrow spectrum antibiotics has been reported to influence the response to oral RV vaccine in humans [151]. In this randomized controlled trial, healthy adults were randomized and administered broad-spectrum (oral vancomycin, ciprofloxacin, metronidazole), narrow-spectrum (vancomycin), or no antibiotics and then vaccinated with oral RV vaccine. Although no difference was observed in anti-RV IgA levels 28 days post-vaccination, the group administered vancomycin revealed slight increase in anti-RV IgA titers 7 days after vaccination. In addition, groups given antibiotics had increased fecal shedding of RV compared to no antibiotic treatment group, which suggest higher RV replication within the intestine [151]. The study provides the first evidence that the gut microbiome has a role to play in RV vaccine immunity in humans. Recently, gut segmented filamentous bacteria (SFB) was found to prevent and cure RV infection in immunodeficient mice [149]. Authors identified a mouse breeding colony that was highly resistant to RV infection and found that resistant mice carried SFB in the microbiota. Co-housing of RV-resistant Rag-1 knock out and RV-susceptible Rag-1 knock out mice, and oral administration of RV-resistant mice feces to RV-susceptible mice resulted in RV-susceptible mice acquiring RV resistance through SFB transfer. It was further revealed that SFB reduces RV infectivity and provide protection by shedding of epithelial cells and replacement with new cells. The results of this study clearly suggest a role of specific gut microbiome in combating RV infections.

To conclude, human data supports a link between microbiome composition and oral RV immunogenicity. However, the association between gut microbiome and porcine rotaviruses has not been studied. Gnotobiotic piglets, because of their close anatomical and physiological resemblance to human infants, have been utilized as models to investigate the effects of nutritional deficiencies, RV infection, and vaccine efficacy in humans [119,156,166,167]. However, the role of gut microbiome in immunity against porcine rotaviruses remains unexplored. We do not know how RV infection in swine affects the structure and functionality of gut microbiome. Given the lack of porcine RVC vaccines, it would be interesting to study the effects of NPE on gut microbiome composition and any association between NPE-induced microbiome changes and RV immune response in gilts and piglets. 

## 7. Overall Conclusions and Future Prospects

Rotavirus infections are endemic in swine herds, and their elimination from swine population is not pragmatic. Moreover, huge genetic diversity and high potential for reassortment adds to the difficulty in developing effective prevention and control strategies. This review mainly focuses on genetic diversity among porcine RVs, immune response against RVs, current methods to stimulate maternal immunity in soon to farrow sows, and the role of the gut microbiome in modulating immune response to RVs. Based on the literature reviewed, the following areas of research have been identified as most important to swine RVs: (1). Routine surveillance of clinical samples from swine herds is needed to identify the effectiveness of current RVA vaccines and emergence of new genotypes due to immune pressure created by use of RVA vaccines. Surveillance data will also help in developing vaccines which are based on prevalent G and P-types in a particular area; (2). The potential of swine enteroids cell cultures to study RVC replication needs to be investigated. Lack of an established cell culture system for RVC hampers the development of an effective RVC vaccine; (3). There is a lack of serological assays to assess antibody response to NPE or natural RV infection which makes it difficult to assess true efficacy of NPE protocols. Earlier, real-time PCR-based assays were used to determine fecal RV shedding as an indirect measure to estimate RV replication and immune response in swine. Hence, it is imperative to develop genotype-specific RVA and RVC specific indirect ELISAs to measure antibody response to RVs in swine; (4). The possibility of a viral vector-based vaccines simultaneously expressing both RVA and RVC protective antigens (VP7 and VP4) needs to be explored for use in swine. This approach could circumvent the need of adapting RVC to cell culture; (5). Currently, NPE material administered to gilts contain both RVA and RVC, but no information is available regarding differences in their capability to colonize swine gut. Hence, it would be interesting to study gut colonization and antibody responses to RVA and RVC in an individual and co-infection swine model; 6. Another area of importance is to characterize gut microbiome changes in swine due to RVs particularly against RVC. Probiotics which reduce the gut colonization by RVCs could be fed to the neonatal piglets to prevent RVC infection in piglets immediately after birth.
